# SARS-CoV-2 virus in raw wastewater from student residence halls with concomitant 16S rRNA bacterial community structure changes

**DOI:** 10.3389/fmicb.2025.1589029

**Published:** 2025-06-02

**Authors:** Ye Li, Kurt T. Ash, Dominique Joyner, Daniel E. Williams, Terry C. Hazen

**Affiliations:** ^1^Department of Civil and Environmental Sciences, University of Tennessee, Knoxville, TN, United States; ^2^Biosciences Division, Oak Ridge National Laboratory, Oak Ridge, TN, United States; ^3^Department of Microbiology, University of Tennessee, Knoxville, TN, United States; ^4^Department of Earth and Planetary Sciences, University of Tennessee, Knoxville, TN, United States; ^5^Institute for a Secure and Sustainable Environment, University of Tennessee, Knoxville, TN, United States

**Keywords:** SARS-CoV-2, COVID-19, raw sewage, 16S rRNA, bacterial community structure

## Abstract

The detection of severe acute respiratory syndrome coronavirus 2 (SARS-CoV-2) RNA in sewage is well-established, but the concomitant changes in microbial compositions during the pandemic remain insufficiently explored. This study investigates the impact of the SARS-CoV-2 virus on microbial compositions in raw sewage, utilizing high-throughput 16S rRNA amplicon sequencing to analyze wastewater samples collected from six dormitories over a one-year field trial at the University of Tennessee, Knoxville. The concentration of SARS-CoV-2 RNA was assessed using a reverse transcription-quantitative polymerase chain reaction. Significant variations in bacterial composition were evident across the six dormitories, highlighting the importance of independently considering spatial differences when evaluating the raw wastewater microbiome. Positive samples for SARS-CoV-2 exhibited a prominent representation of exclusive species across all dormitories, coupled with significantly reduced bacterial diversity compared to negative samples. The correlation observed between the relative abundance of enteric pathogens and potential pathogens at sampling sites introduces a significant dimension to our understanding of COVID-19, especially the notable correlation observed in positive SARS-CoV-2 samples. Furthermore, the significant correlation in the relative abundance of potential pathogens between positive and negative SARS-CoV-2 raw sewage samples may be linked to the enduring effects of microbial dysbiosis observed during COVID-19 recovery. These findings provide valuable insights into the microbial dynamics in raw sewage during the COVID-19 pandemic.

## Introduction

Although sewage is often assumed to mirror the human gut microbiome, raw sewage samples exhibit notable microbial differences, indicating substantial contributions from non-human sources. The microbial composition of sewage, while partially originating from the human gut, includes a diverse array of both beneficial and pathogenic species, with bacteria and viruses playing central roles (Newton et al., 2015). Although robust evidence supports some similarity between the microbial profiles of raw sewage and the human gut, quantitative analyses suggest that the overlap is limited. The total abundance of high-level genera in influent sewage has been estimated at nearly 50%, a proportion similar to that observed in the human gut, indicating stool as a major contributor to sewage microbiota (Cai et al., 2014). However, sequences comprising approximately 78% of a stool sample account for only about 12% of a sewage sample; when extrapolated, this suggests that only 15% of amplicons in raw sewage originate from human stool (Newton et al., 2015). Additionally, fecal-derived bacteria have been found to make up a relatively small fraction of taxa in collected sewage samples, emphasizing the substantial influence of environmental sources (Fierer et al., 2022). These findings raise questions about whether raw sewage accurately reflects the microbial composition of the human gut.

Crucially, COVID-19–related respiratory infections have been linked to alterations in gut microbiota composition (Gu et al., 2020; Zuo et al., 2020). The dysbiosis of COVID-19 may enhance gut permeability, leading to secondary infections and organ failure. Simultaneously, disruptions in gut barrier integrity could facilitate the translocation of SARS-CoV-2 from the lungs to the intestinal lumen (Aktaş and Aslim, 2020). Gu et al. (2020) and Zuo et al. (2020) observed that, compared to fecal samples from healthy people, fecal samples from COVID-19 patients had significantly reduced bacterial diversity, a significantly higher relative abundance of opportunistic pathogens and a lower relative abundance of beneficial symbionts. Liu et al. (2022) even found that gut dysbiosis persisted even after clearance of SARS-CoV-2 at 6 months. Patients with COVID-19 exhibit significant alterations in fecal microbiomes, suggesting potential changes in the wastewater microbiome during the pandemic. Currently, research on microbial compositions in wastewater with positive and negative SARS-CoV-2 samples remains limited, with Gallardo-Escárate et al. (2021) being the sole study to explore such dynamics across three sampling communities using nanopore technology. Their findings highlighted a robust association between the microbiota of positive SARS-CoV-2 wastewater samples and enteric bacteria. Notably, integrating the Wastewater-Based Epidemiology tool with metagenomic analysis, employing 16S rRNA sequencing technology to investigate changes in sewage microbiota during the COVID-19 pandemic, remains an unexplored avenue that warrants further research.

This study employed 16S rRNA sequencing to analyze microbial compositions in raw sewage samples, differentiating between those with positive and negative COVID-19 status. The primary goal was to identify distinct patterns or shifts in the bacterial community associated with the presence of the virus. Through the utilization of this technology, the research aimed to provide a nuanced understanding of the dynamics of viral shedding, microbial interactions, and the overall impact of SARS-CoV-2 on the sewage microbiome over a year-long field trial conducted in six campus dormitories. Including COVID-19-negative sewage samples as a control allowed for identifying specific changes attributable to viral presence, facilitating the establishment of correlations between the sewage microbiota and COVID-19 prevalence in human communities.

## Results

### Concentration of SARS-CoV-2 in raw sewage

**Figure 1:**
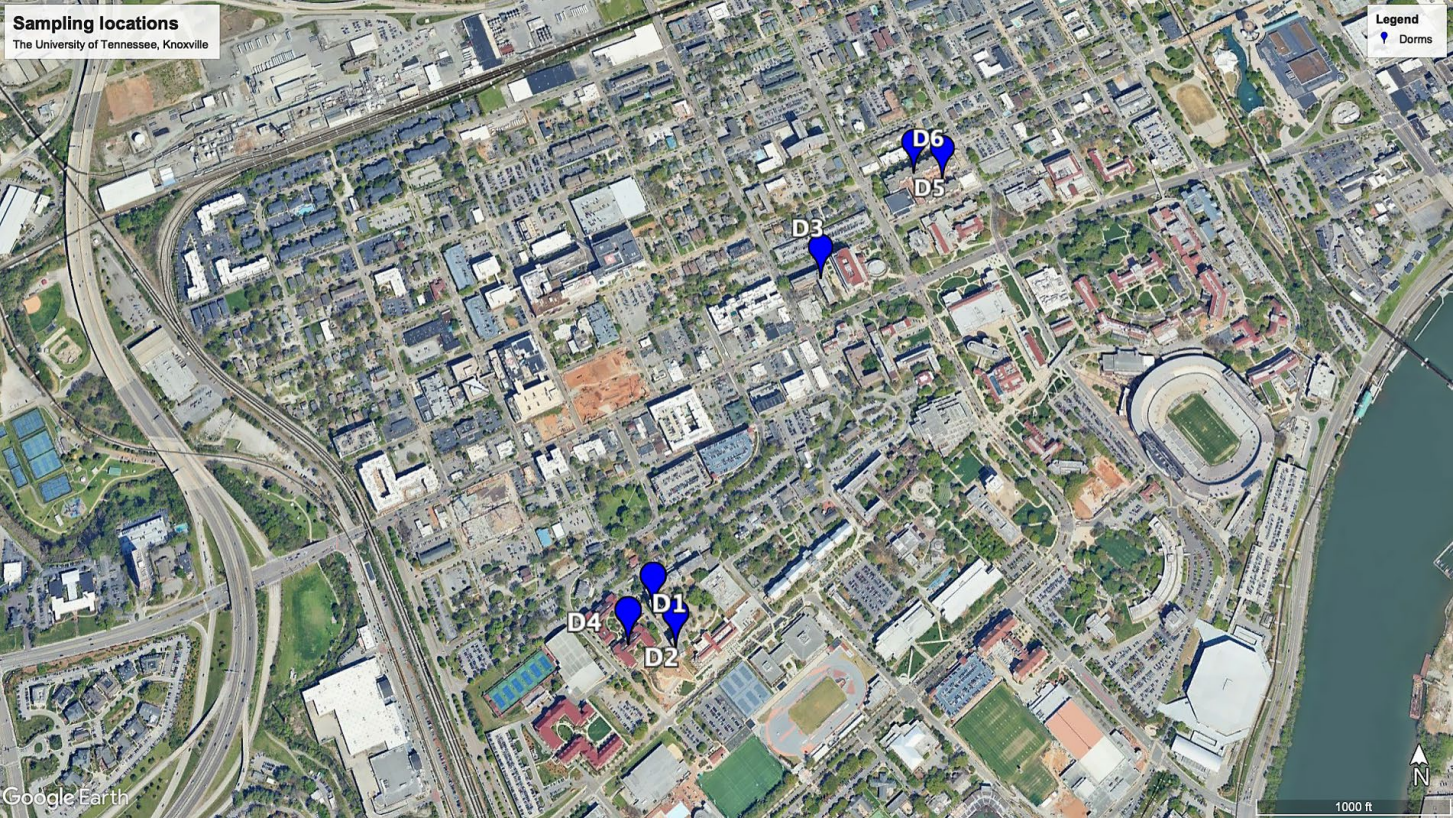
Map of the sampling locations on the University of Tennessee-Knoxville campus.

**Figure 2:**
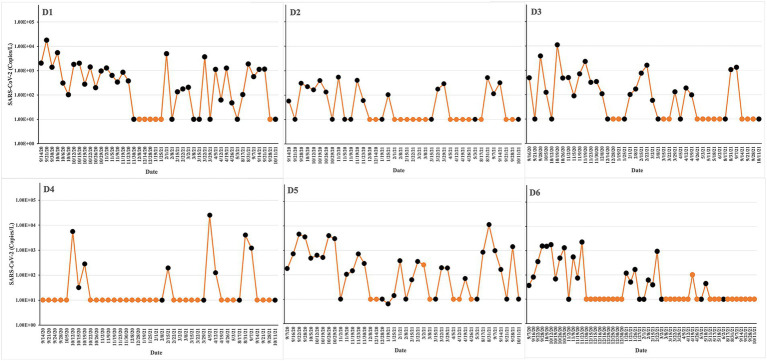
Experimental design and sampling points/times for microbiome sequencing. SARS-CoV-2 concentrations are indicated as yellow lines. The yellow points at 10 copies/L represent negative samples. The sequencing runs are indicated as black points. The virus load was estimated by qPCR in untreated wastewater from different dormitories: D1, D2, D3, D4, D5, and D6. The study was conducted from Sep 2020 to Oct 2021.

The 174 raw sewage samples included in this study were collected from 6 different dormitories in the same sewage network across the University of Tennessee, Knoxville (Figure 1). Concentrations of SARS-CoV-2 from September 2020 to October 2021 within various high-density student residence halls ranged from 2.02 ± 2.19 to 3.09 ± 3.46 log10 copies/L (Figure 2). Over the sampling period, SARS-CoV-2 concentrations were consistently measured at different levels in the respective halls: 3.09 ± 3.46 log10 copies/L in D1, 2.02 ± 2.19 log10 copies/L in D2, 2.80 ± 3.26 log10 copies/L in D3, 2.97 ± 3.61 log10 copies/L in D4, 2.94 ± 3.30 log10 copies/L in D5, and 2.36 ± 2.71 log10 copies/L in D6.

Positive rates, calculated by dividing the number of positive samples by the total number of samples and multiplying by 100%, varied across the halls. Specifically, the positive rates were 70% in D1, 39% in D2, 52% in D3, 20% in D4, 68% in D5, and 37% in D6 (Table 1).

**Table 1:** Raw wastewater data information for D1, D2, D3, D4, D5, and D6.

Dorms	pH	Positive Rate	Relative abundance of potential pathogen	
			Positive SARS-CoV-2 Sample	Negative SARS-CoV-2 Sample
D1	6.71–9.08	70.00%	40.04%	40.35%
D2	6.83–8.27	39.00%	21.80%	29.37%
D3	6.51–8.97	52.00%	17.58%	13.88%
D4	6.27–9.01	20.00%	30.63%	24.11%
D5	6.38–8.95	68.00%	43.49%	45.17%
D6	5.72–8.63	37.00%	41.30%	45.64%

### Characteristics of the predominant flora in different dormitories

#### Characterization on phylum, family, and genus level

**Figure 3:**
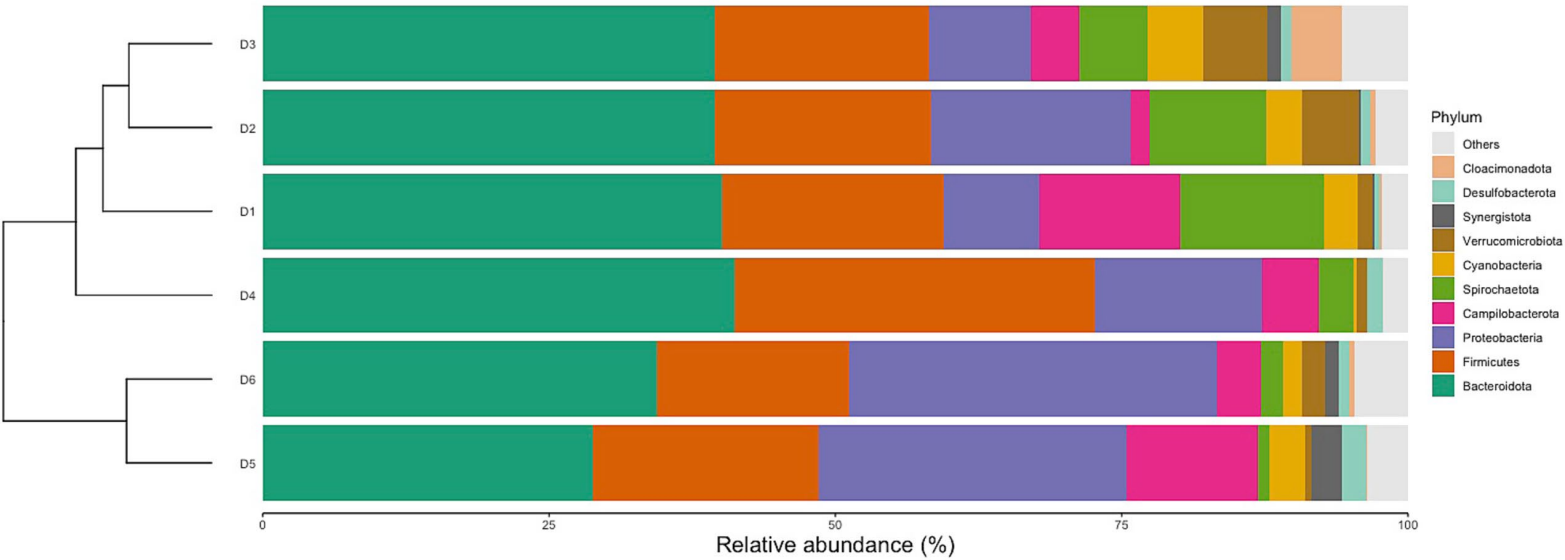
Relative abundances of the top 10 dominant phyla in 6 dormitories.

Classification of reads revealed 56 phyla, 145 classes, 315 orders, 548 families, and 1,170 genera. The relative abundances of the top 10 phyla varied between dormitories (Figure 3). The phylum Bacteroidetes was the most abundant across all sampling sites, with relative abundance ranging from 46 to 27%. Firmicutes were the second most abundant phylum in dormitories 1, 2, 3, and 4, with relative abundance varying from 35 to 15%. Meanwhile, Proteobacteria was the second most abundant phylum in dormitories 5 and 6, with relative abundance ranging from 41 to 25%. Additionally, dormitories 2 and 3 have the same four highest abundance phyla of Bacteroidetes, Firmicutes, Proteobacteria, and Spirochaetota. Similarly, Dormitories 5 and 6 have the same four highest abundance phyla of Bacteroidetes, Proteobacteria, Firmicutes, and Campilobacterota.

**Figure 4:**
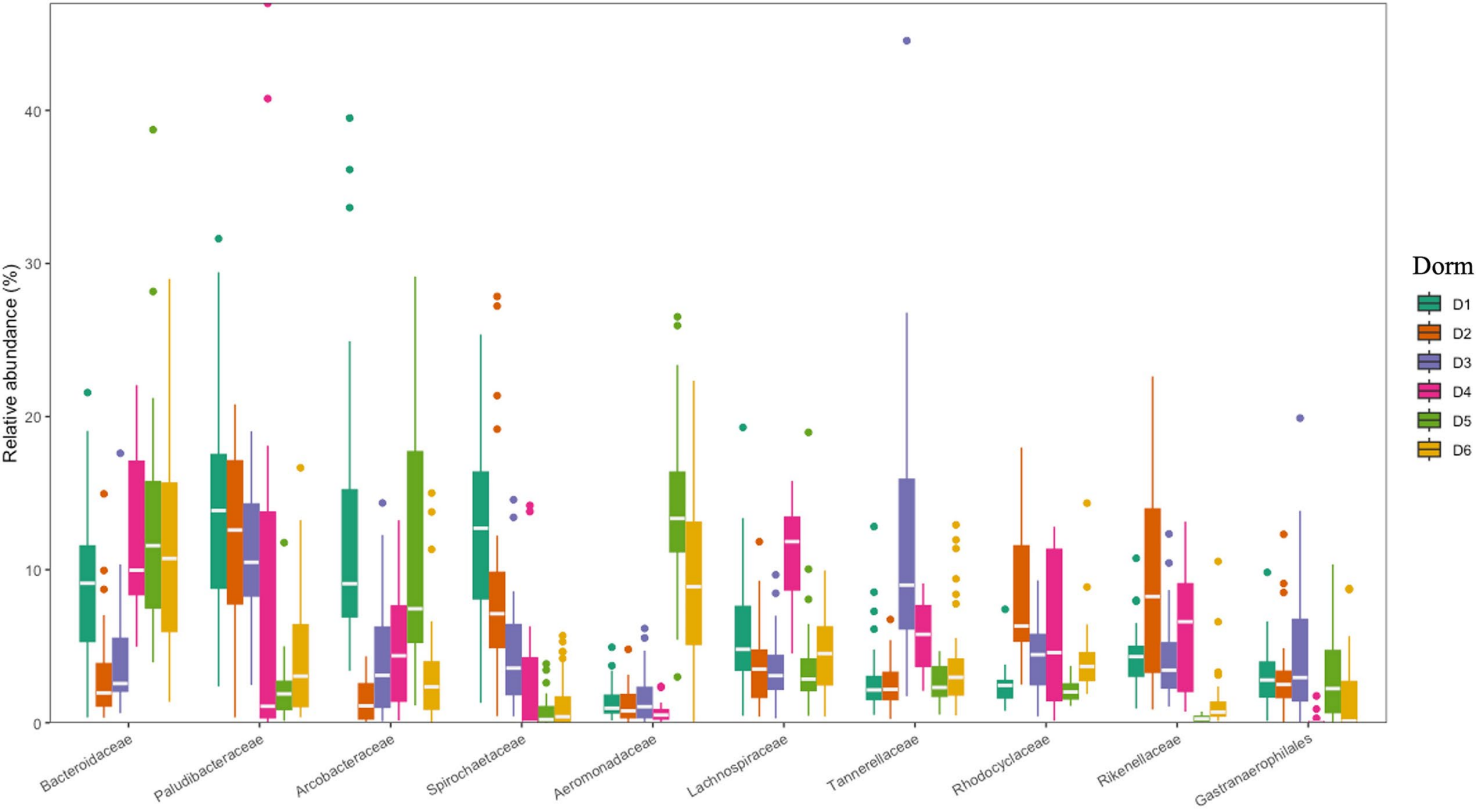
Relative abundances at family levels for six dormitories.

The 10 most dominant families showcasing significant differences among the six dormitories (Figure 4). Paludibacteraceae and Spirochaetaceae were the dominant families, with varying relative abundance in D1, D2, and D3. Bacteroidaceae dominated libraries generated from D4, D5, and D6, with distinct relative abundance up to 13%.

**Figure 5:**
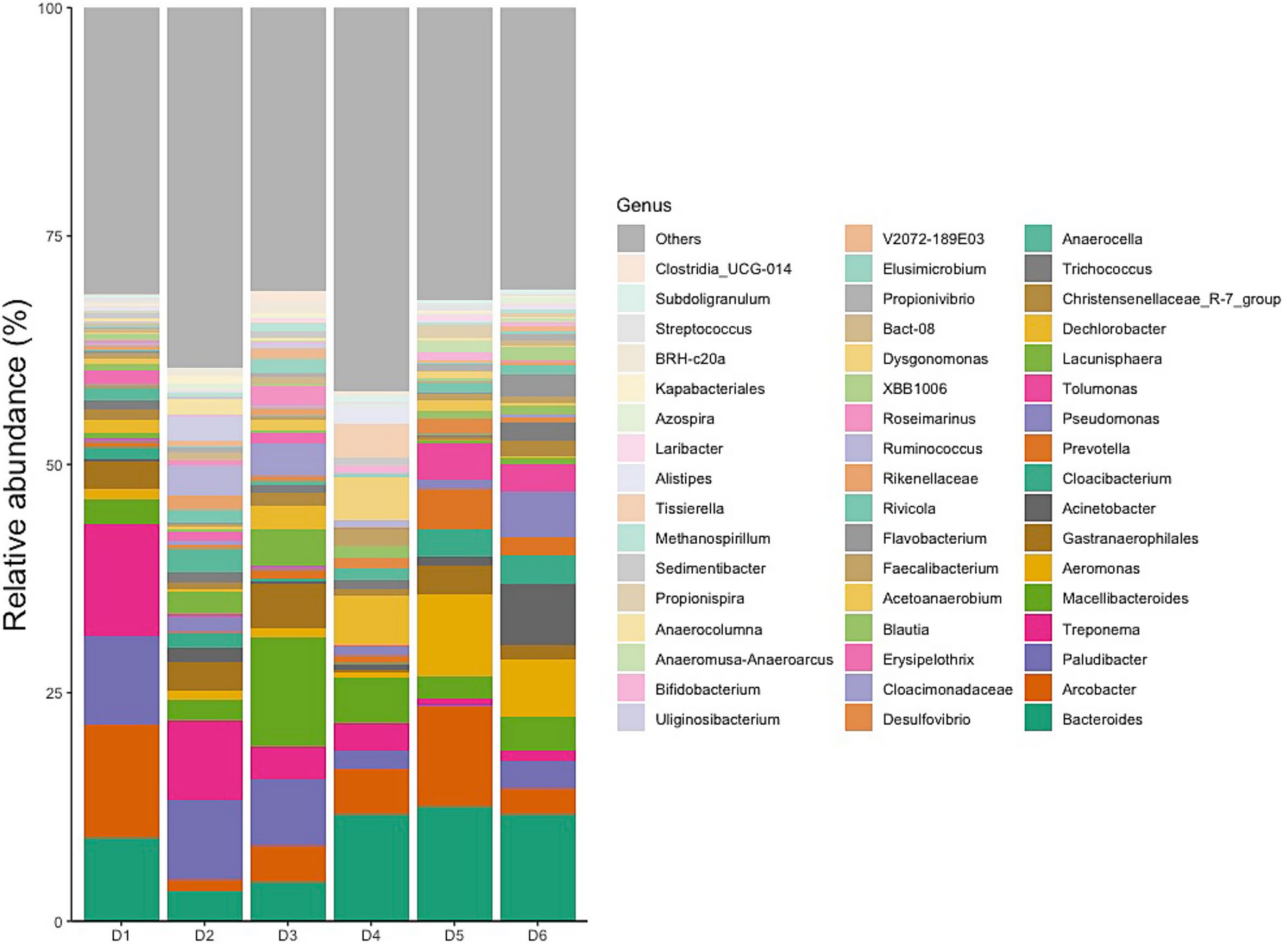
Relative abundances of top 50 genera.

Typical gut bacteria were also found at very high levels in the sewage such as Bacteroides, Acinetobacter, Prevotella, Pseudomonas, Blautia, Faecalibacterium, Ruminococcus, and Dorea, corresponding to ranks 1, 8, 10, 11, and 21 within the top 50 genera in Figure 5 (Furet et al., 2009; Cai et al., 2014; Bäckhed et al., 2015; Do et al., 2019). Among the top 50 genera, 14 genera (34%) were identified as potential pathogens, including Bacteroides, Arcobacter, Treponema, Aeromonas, Acinetobacter, Prevotella, Pseudomonas, Erysipelothrix, Faecalibacterium, Flavobacterium, Ruminococcus, Bifidobacterium, Laribacter, and Streptococcus (Cai and Zhang, 2013; Cai et al., 2014; Do et al., 2019; Oluseyi Osunmakinde et al., 2019; Poopedi et al., 2023).

Dormitories D1 to D6 exhibited a varying relative abundance of potential pathogens, with 13 (40%), 12 (23%), 11 (18%), 12 (28%), 14 (45%), and 14 (41%) genera recognized as such, respectively (Figure 5). Notably, a substantial number of these potential pathogens displayed an increased relative abundance in samples from D1, D5, and D6 compared to other sites. Among the detected enteric pathogens in the top 50 genera were *Arcobacter*, *Aeromonas*, and *Laribacter*, with total relative abundances of 14, 2, 5, 6, 21, and 9% from D1 to D6, respectively. *Arcobacter* and *Aeromonas* were identified across all six dormitories, while *Laribacter* was exclusively found in D3, D5, and D6. Relative abundance of enteric pathogens and potential pathogens correlated significantly (Pearson Correlation = 0.842, *p* = 0.018). The genus Mycobacterium, which encompasses potential respiratory tract-associated pathogens, comprised between 0.02 and 0.15% of the total bacterial community across all six dormitories.

### Diversity of bacterial communities

**Figure 6:**
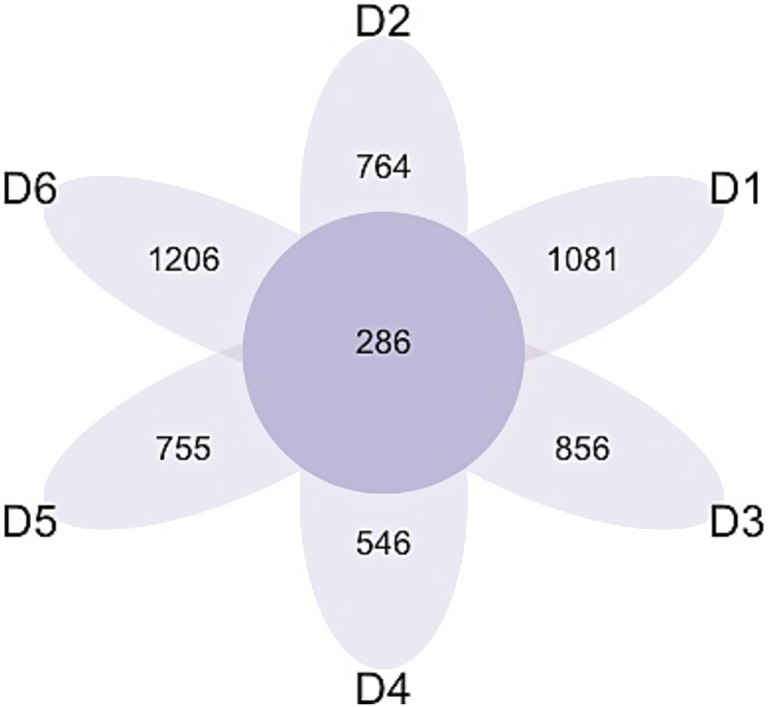
Venn diagram of exclusives and shared bacteria among the 6 dormitories.

The analysis of the microbiota communities within the collected wastewater samples revealed significant distinctions across all sampled locations (Figure 6). At the species level, dormitory 6 (D6) exhibited the highest count of exclusive taxa, totaling 1,206, while the other dormitories (D1 to D5) displayed varying counts of exclusive taxa, ranging from 546 to 1,081 species. A core microbiome consisting of 286 bacterial species was consistently observed across all sampled dormitories.

**Figure 7:**
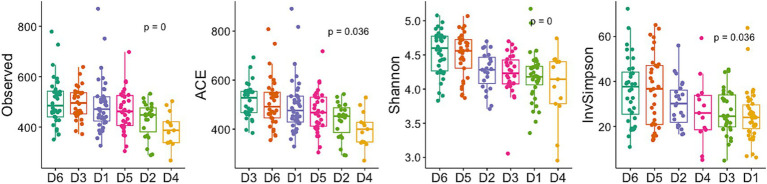
Diversity index in 6 dormitories. The box-and-whisker plots show the mean (diamond), median (middle bar), first quartile (lower bar), third quartile (upper bar), minimum observation above the lowest fence (lower whisker), and maximum observation below the upper fence (upper whisker) of common α-diversity metrics for each group. The *p* values for the comparison between groups using linear regression models including semester as covariate is also shown.

**Figure 8:**
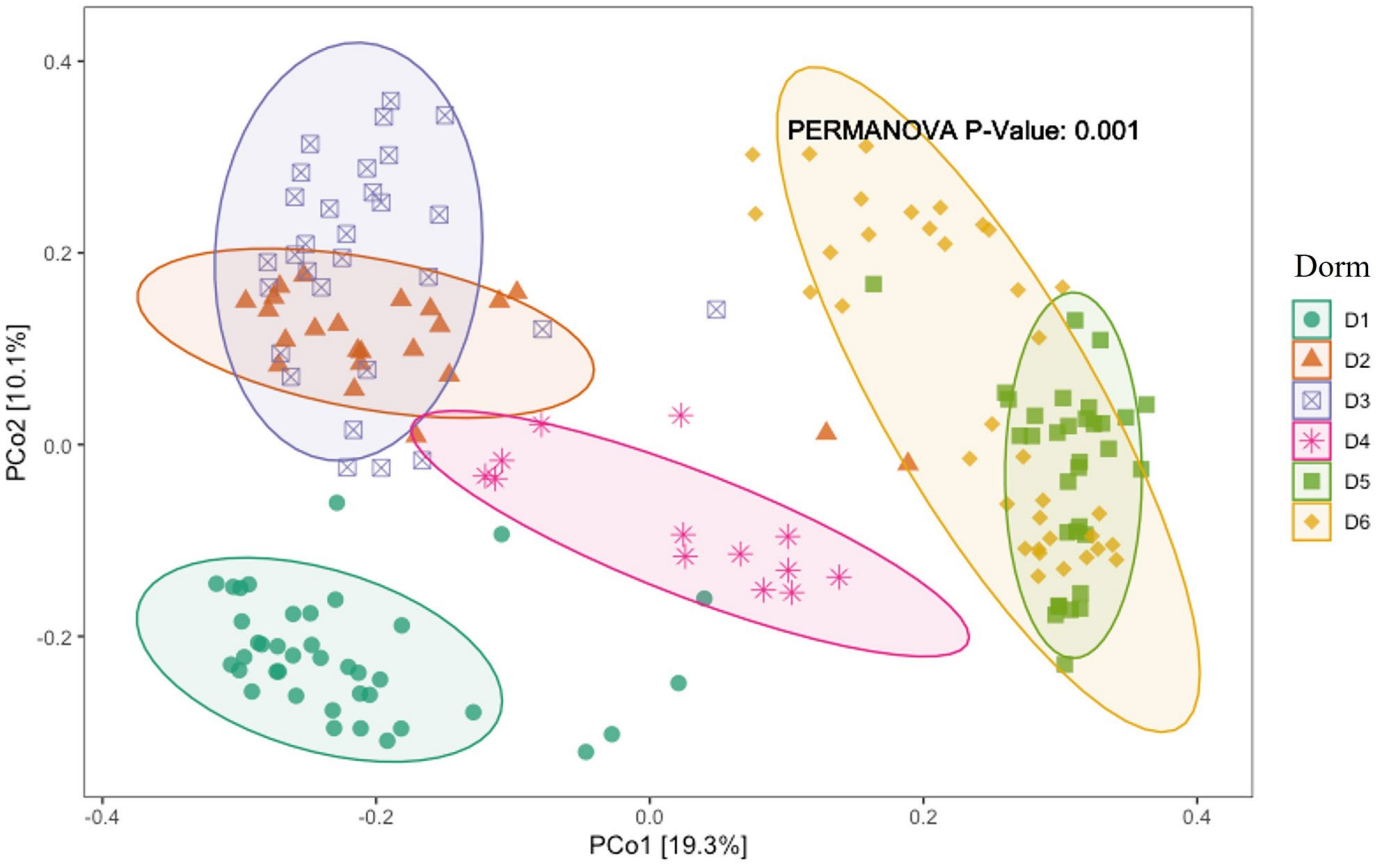
The scatter plots show each participant’s microbial community composition (small circles) by group, as well as each group’s centroid (large circles) and 95% CI ellipses. The scatter plots were generated using Principal Coordinates Analysis (PCoA) ordination based on common β -diversity metrics. For ease of visualization, only 2 dimensions were used. The p values for the comparison between groups using permutational multivariate ANOVA models including semester as covariate is also show.

Alpha diversity differed significantly between dormitories (Figure 7). The highest diversity was observed in D6 across the diversity indices of observed, Shannon, InvSimpson, and Fisher. Beta diversity differed significantly between dormitories (Figure 8), as assessed through Bray-Curtis distance metrics. Clustering analysis revealed distinct groupings, and a permutational multivariate ANOVA, with semester as a covariate, showed significant differences in β-diversity between dormitories (*p* < 0.05).

### Characteristics of the predominant flora in positive and negative samples

#### Characterization on phylum, family, and genus level

**Figure 9:**
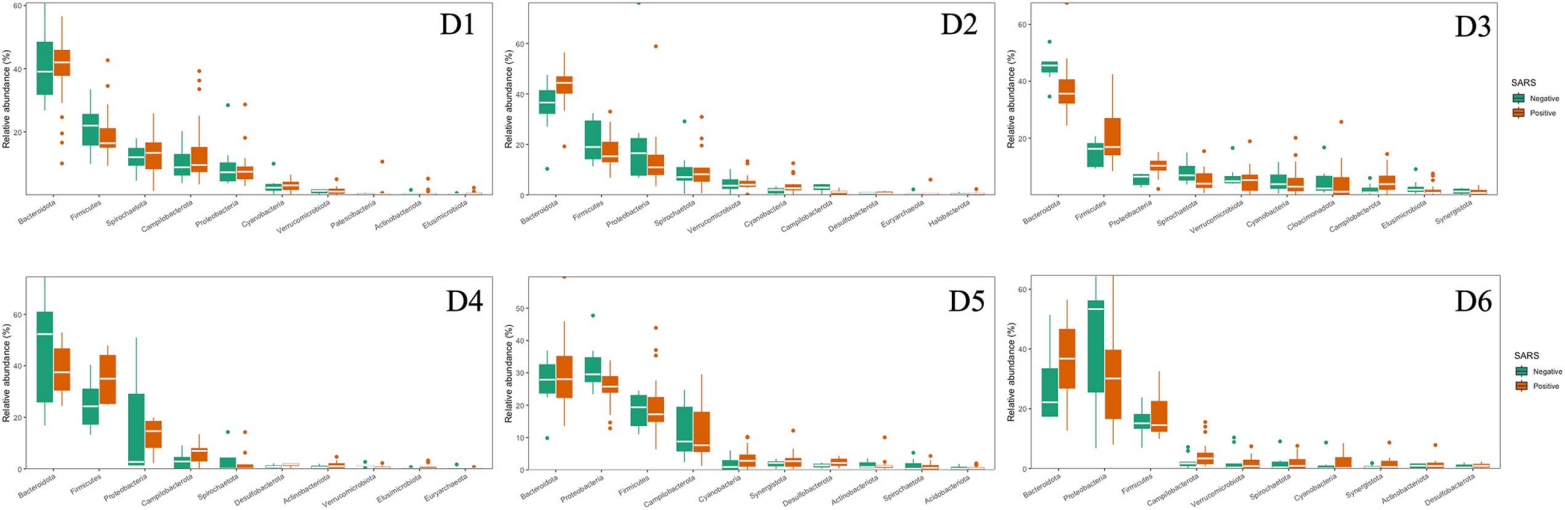
Relative abundances of the top 10 dominant phyla in 6 dormitories with positive and negative SARS-CoV-2 samples.

**Figure 10:**
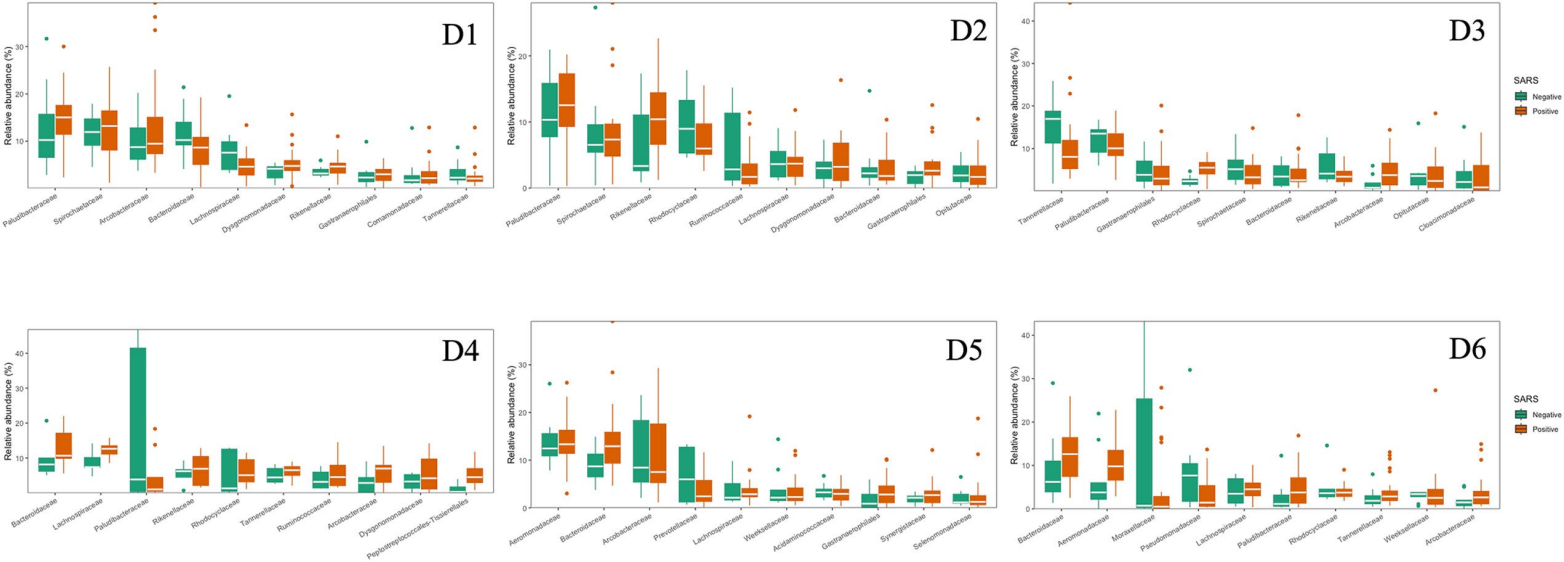
Relative abundances of the top 10 dominant family in 6 dormitories with positive and negative SARS-CoV-2 samples.

**Figure 11:**
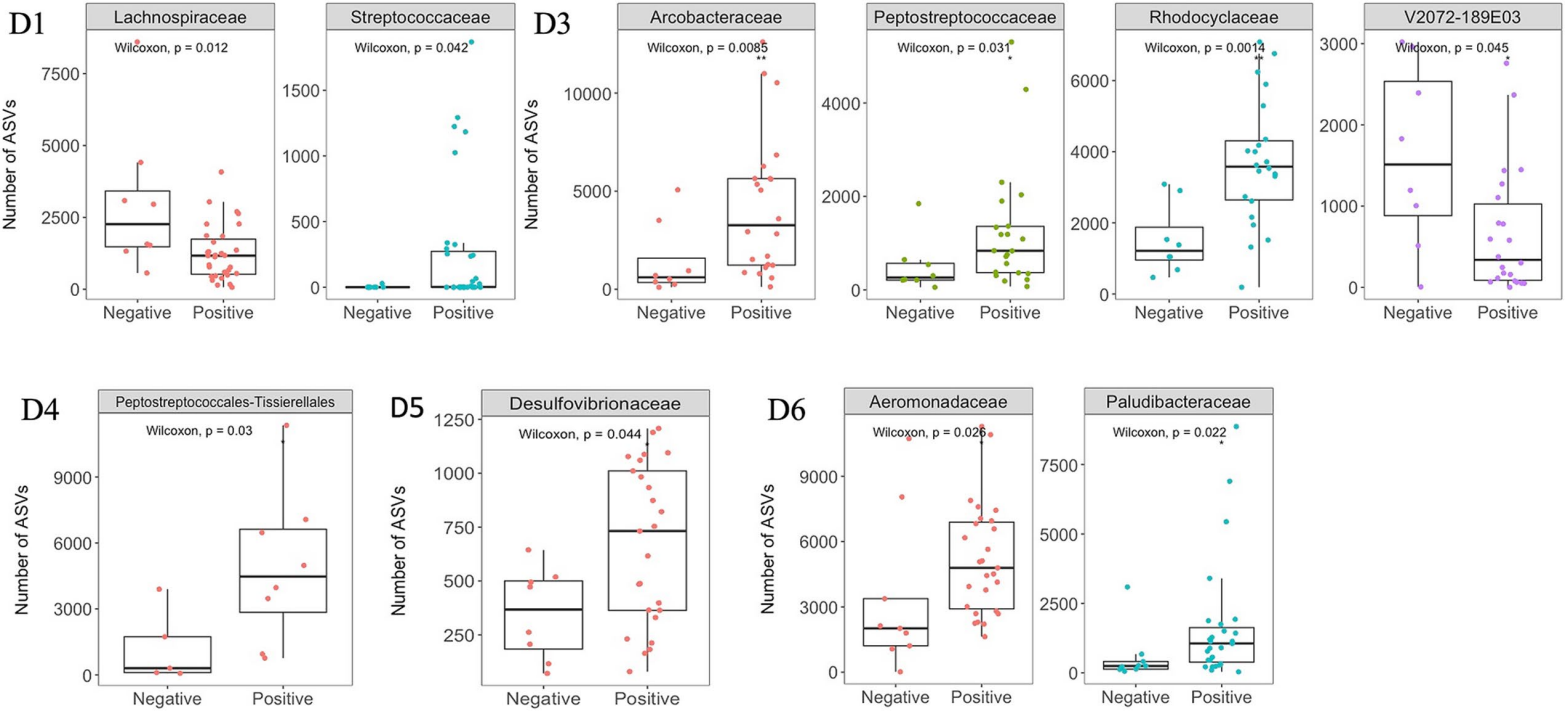
Significant changes at the family level between SARS-CoV-2 positive and negative samples across six dormitories. Y-axis represents the number of ASVs (Amplicon Sequence Variants) associated with each bacterial family.

The dominant phyla and families were consistent at different dormitories, regardless of the COVID-19 status (Figures 9, 10). However, some low abundance families unveiled several noteworthy distinctions within the dormitories (Figure 11). Dormitory 1 showed a distinction between the families Lachnospiraceae and Streptococcaceae. Similarly, dormitory 3 exhibited significant differences in the relative abundances of Arcobacteraceae, Peptostreptococcaceae, Rhodocyclaceae, and V2072-189E03 between COVID-19 positive and negative samples. In dormitory 4, Peptostreptococcales-Tissierellales displayed significant variation in relative abundances between the two sample groups. In dormitory 5, Desulfovibrionaceae demonstrated a significant difference in relative abundances based on COVID-19 status. Lastly, in dormitory 6, significant differences were observed in the relative abundances of Aeromonadaceae and Paludibacteraceae between COVID-19 positive and negative samples.

LEfSe analysis did not identify biomarkers associated with COVID-19 status across the six dormitories (Supplementary Figure S1). Instead, distinct biomarkers were exclusively found in dormitories 3, 4, and 5, suggesting unique microbial signatures associated with COVID-19 status in these specific dormitory environments.

**Figure 12:**
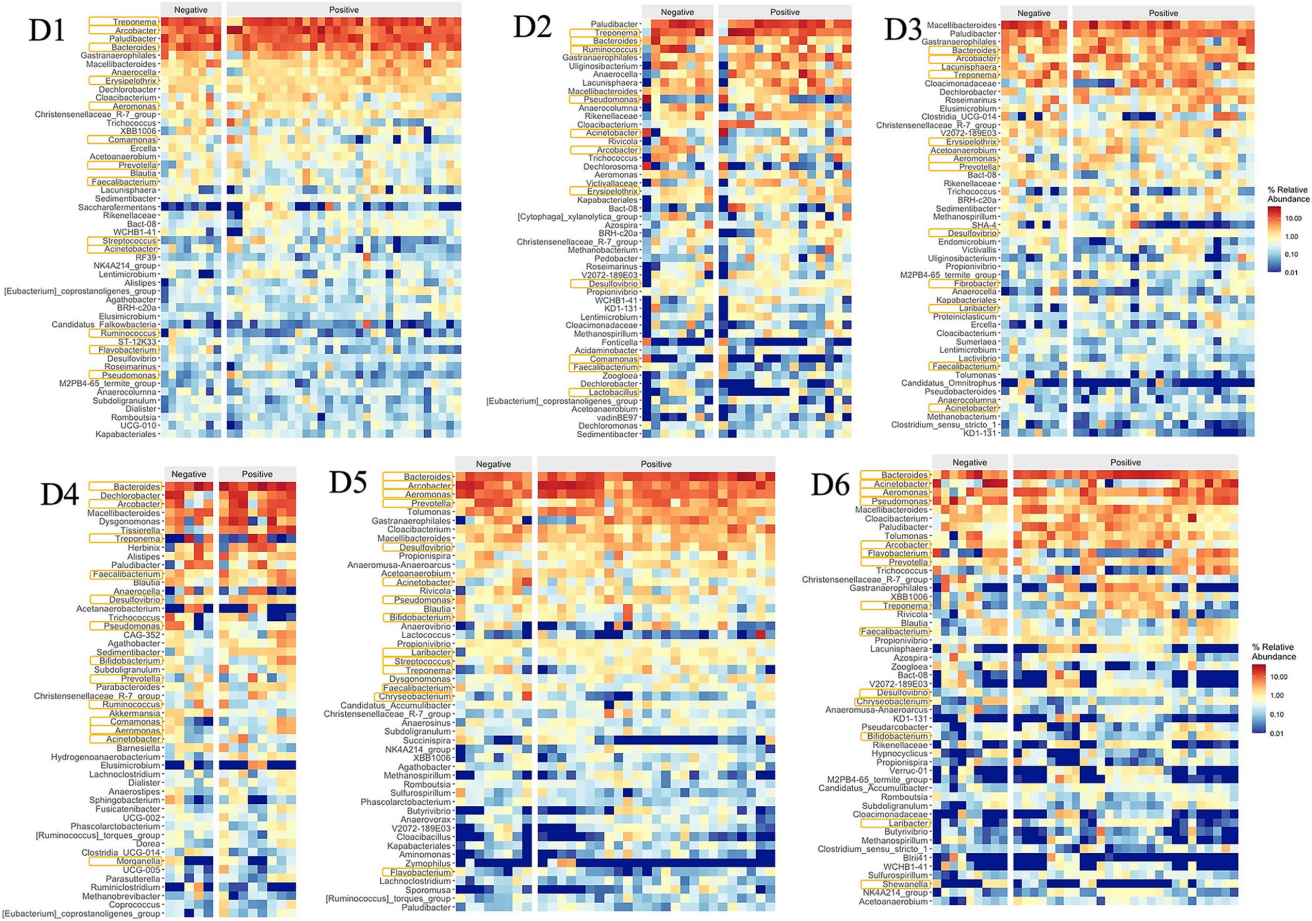
Relative abundances of top 50 genera and potential pathogens with positive and negative SARS-CoV-2 samples in 6 dormitories. The genera are listed from the highest relative abundance (top) to the least relative abundance (bottom). The pathogens are marked with an orange box around their name.

The relative abundance of potential pathogens correlated significantly between SARS-CoV-2 positive and negative samples (Pearson correlation coefficient = 0.918, *p* = 0.010, Table 1). Additionally, the relative abundance of enteric pathogens and potential pathogens correlated significantly in SARS-CoV-2 positive samples (Pearson correlation coefficient = 0.817, *p* = 0.024), independent of the relative abundance of potential pathogens in SARS-CoV-2 negative samples (Figure 12).

### Diversity of bacterial communities

**Figure 13:**
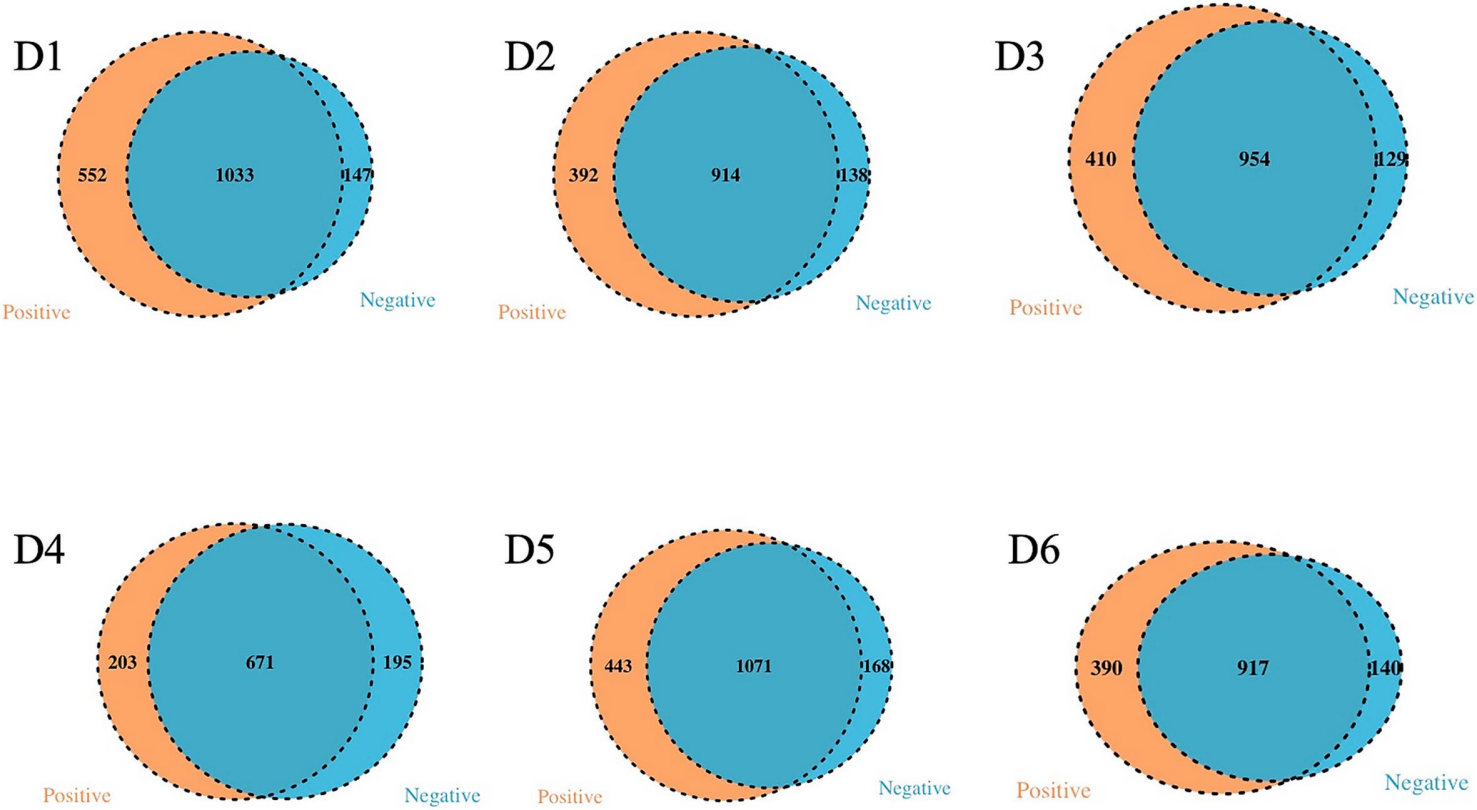
Venn diagram of exclusives and shared bacteria with positive and negative SARS-CoV-2 samples in the 6 dormitories.

Samples testing positive for SARS-CoV-2 demonstrated a higher diversity of taxa compared to their negative counterparts (Figure 13). Exclusive species were most prominently represented in positive samples for SARS-CoV-2 collected from D1 at 32%, while D3 exhibited the lowest representation at 19%. Conversely, negative samples for SARS-CoV-2 were associated with exclusive bacterial species in wastewater collected from D3 (18%), with D1 displaying the lowest representation at 8%. Despite the SARS-CoV-2 status, the analysis further indicated a low representativity for exclusive bacteria found in other dormitories.

An observation revealed a correlation between the positive rate of sampling sites and the relative abundance of exclusive species in positive samples (Pearson Correlation = 0.771, *p* = 0.036). Additionally, 1,033, 914, 954, 671, 1,071, and 917 taxa were present in both SARS-CoV-2 positive and negative samples from D1 to D6, respectively.

**Figure 14:**
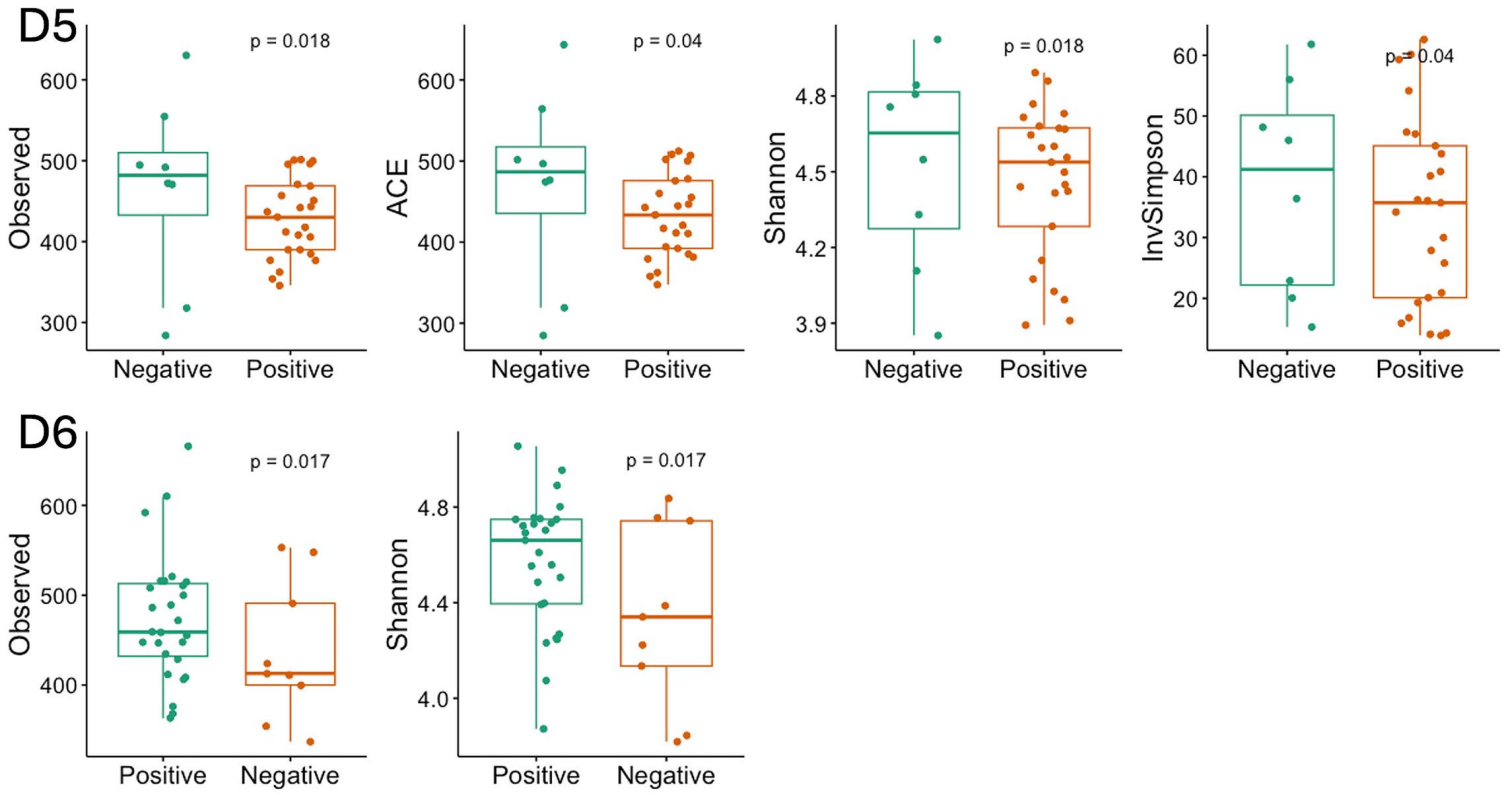
Diversity index with significant difference between the positive and negative SARS-CoV-2 samples in D5 and D6. The box-and-whisker plots show the mean (diamond), median (middle bar), first quartile (lower bar), third quartile (upper bar), minimum observation above the lowest fence (lower whisker), and maximum observation below the upper fence (upper whisker) of common α-diversity metrics just for significant group. The p values for the comparison between groups using linear regression models including semester as covariate is also shown.

**Figure 15:**
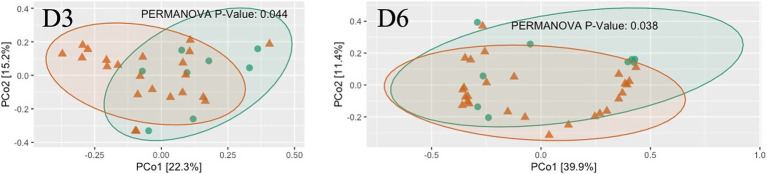
The scatter plots show each participant’s microbial community composition (small circles) by D4 and D6, as well as their centroid (large circles) and 95% CI ellipses. The scatter plots were generated using Principal Coordinates Analysis (PCoA) ordination based on common b-diversity metrics. For ease of visualization, only 2 dimensions were used. The *p* values for the comparison between groups using permutational multivariate ANOVA models including semester as covariate is also shown.

The α-diversity of the microbiome across all locations exhibited a general trend of being higher in negative samples compared to positive samples. Specifically, the observed species index showed a significant difference in D5 and D6 (*p* < 0.05, Figure 14), as determined through linear regression models that incorporated semester as a covariate. Notably, significant differences in the measured *β*-diversity metrics were discerned in D3 and D6 between groups (p < 0.05 for the Bray-Curtis indices, using permutational multivariate ANOVA with semester as a covariate), as illustrated in Figure 15.

## Discussion

Dormitory (as a proxy for geographic location or shared living environment) has a stronger impact on microbial diversity than COVID status, and we did not detect a statistically significant interaction between the two factors (Table 2). Therefore, our analysis supports the interpretation that the dormitory effect can be evaluated independently.

**Table 2:** Two-way analysis of variance of beta diversity with dorm and SARS-CoV-2.

Source	Df	Sum of squares	R2	*F*	*p*
Dorm	5	20.86	0.398	22.37	0.001
SARS	1	0.24	0.005	1.28	0.163
Dorm: SARS	5	1.08	0.021	1.16	0.143
Residual	162	30.22	0.577		

Our study did not unveil a significant universal biomarker distinguishing positive from negative SARS-CoV-2 sewage samples across all sampling locations. This contrasts with findings showing that specific microbial biomarkers can distinguish COVID-19 patients from healthy individuals (Gu et al., 2020). However, the absence of detectable SARS-CoV-2 in some patients does not necessarily indicate full recovery of the gut microbiota. Microbial restoration may require an extended period, even after viral clearance. Persistent dysbiosis following infection has been reported, with reduced bacterial diversity and richness observed up to 3 months post-infection compared to healthy controls (Zhang et al., 2023). This reduction was accompanied by a lower abundance of beneficial commensals and a higher abundance of opportunistic pathogens. Hence, the significant correlation in the relative abundance of potential pathogen between positive and negative SARS-CoV-2 sewage samples in our study may be attributed to the lingering effects of microbial dysbiosis observed in COVID-19 recovery.

The identified variations at the phylum, family, and genus levels across the six dormitories shed light on the geographic differences in bacterial composition in this study (Figures 3–5). The analysis revealed two clusters of community types, as illustrated in Figure 3. Dormitories 2, 3, 1, and 4, organized by the closer relationship of bacterial phyla in each building, exhibited similar dominance patterns in these phyla, while D5 and D6 exhibited comparable compositions. The spatial arrangement depicted in the map (Figure 1) highlights that D1, D2, and D4 are in proximity, D5 and D6 are likewise nearby, and D3 is closer to D5 and D6. This spatial variation suggests a potential impact of geographic factors on the microbial composition in different dormitories. This observation aligns with the study by Fierer et al. (2022) finding five clusters of 17 different locations, revealing no strong relationship with the distance between sampling locations.

The significant alpha and beta diversity further underscore pronounced geographical variations in microbial communities in this study, aligned with Fierer et al. (2022) findings. Their emphasis on independently considering spatial variations when assessing the wastewater microbiome highlights the need to account for the influence of location on microbial diversity. Their research identified geographic variations in bacterial composition unrelated to sewer material, sewer depth, or resident human population on the campus. They attributed these variations to sample pH, with total suspended solids concentrations and sample volume playing a lesser role. This pH correlation aligns with studies by Fujii et al. (2012) and Lindström et al. (2005), which demonstrated the close association between pH and shifts in bacterial community composition in aquatic environments. Despite the detected variations in bacterial composition across dormitories in our study, the pH did not exhibit significant changes. Future research could explore specific factors such as organic carbon or nutrient concentrations to better understand the observed geographic variations in microbial communities.

The analysis of the microbial community in raw sewage yielded results consistent with previous research, indicating the influence of the human gut bacterial community on the bacterial profile in raw sewage. Specifically, the phyla Bacteroidota was identified as the most abundant and variable across samples, aligning with findings from Arumugam et al. (2011). However, a study by Cai et al. (2014) reported Firmicutes as the most dominant phylum in influent samples, asserting its alignment with the human microbiome composition. The findings of Turnbaugh et al. (2006) and Clemente et al. (2012) clarify this discrepancy indicating that the gut microbiota typically showcases dominance of bacteria, particularly from the Bacteroidota and Firmicutes divisions. Furthermore, Huttenhower et al. (2012) revealed that gut microbiota relationships were characterized by inverse associations with Bacteroidota, varying from dominance in some subjects to a minority in others with a greater diversity of Firmicutes. These nuanced observations highlight the intricate dynamics of the human gut microbiota and underscore the pivotal roles played by Bacteroidota and Firmicutes in shaping microbial profiles observed in raw sewage.

The significant differences in bacterial composition observed across the six dormitories prompted a recommendation for separate analyses of the 16S rRNA data for each dormitory. This approach aims to mitigate biases that may arise when combining data from diverse dormitory settings. The prominent representation of exclusive species in positive samples for SARS-CoV-2 were found across all six dormitories supports the findings of Gallardo-Escárate et al. (2021). Moreover, the observed trend of higher α-diversity in the microbiome of negative samples compared to positive samples across some locations echoes the results reported by Gu et al. (2020) and Yeoh et al. (2021), who documented a significant decrease in gut microbiota diversity and abundance in COVID-19 patients relative to healthy individuals.

The observed correlation between the relative abundance of enteric pathogen and potential pathogens at sampling sites adds a significant layer of understanding in the context of COVID-19, particularly highlighting the notable association between the relative abundance of enteric pathogen and potential pathogen in positive SARS-CoV-2 samples. The presence of three enteric genera, namely, *Arcobacter*, *Aeromonas*, and *Laribacter*, in our study, commonly residing in the human intestines and potentially utilizing pathogenic mechanisms to induce gastrointestinal tract infections, emphasizes the relevance of these microbes in the sewage context during the pandemic. Notably, the *Aeromonas* genus ranked as the third leading cause of diarrhea after Campylobacter and Salmonella, exhibited a notably high abundance exclusively in D5 (9%) and D6 (6%) compared to other dormitories, where the abundance ranged from 0.66 to 1.14%. Additionally, two *Arcobacter* species, *A. butzleri, and A. cryaerophilus,* are considered emerging pathogens posing threats to human health*, adding* depth to discussing potential pathogenic risks in the sewage microbiome. Additionally, the genus *Laribacter*, represented by the species *L. hongkongensis*, known for its associations with traveler gastroenteritis and diarrhea (Beilfuss et al., 2015), further contributes to understanding the microbial landscape in the context of COVID-19.

The low abundance of *Mycobacterium* in our samples aligns with findings from previous studies, providing a basis for comparative analysis. The genus Mycobacterium has been consistently detected at very low abundances in wastewater. In both influent and effluent samples, its overall abundance remained below 0.02% (Cai and Zhang, 2013). Similarly, Mycobacterium was observed exclusively in October effluent samples, also at a relative abundance below 0.02% (Numberger et al., 2019; Aktaş and Aslim, 2020). The 16S rRNA gene sequences analysis in our work determined the presence of the bacterial genera but not species. These genera may contain both pathogenic and non-pathogenic species. Therefore, the identification of pathogens requires further study.

## Materials and methods

### Raw sewage sampling and sample processing

Raw wastewater was systematically collected from six student residence halls on the University of Tennessee, Knoxville campus, as illustrated in Figure 1. Each of these residential dormitories accommodated a population of over 400 students, and a detailed summary of their characteristics is presented in Table 3. Sampling was from access points to the main sewage pipe in the basement of the building or at the first access point to a raw sewer manhole immediately outside the building, specifically before the convergence or merging with other sewer conduits. This sampling initiative occurred from September 14, 2020, to October 11, 2021.

**Table 3:** Demography data for D1, D2, D3, D4, D5, and D6.

Sampling site	Sampling point	Gender	Student Number
D1	Direct Dispense from the valve	Male	387–504
D2	Direct Dispense from the valve	Female	469–531
D3	Direct Dispense from the valve	Mix	254–279
D4	Direct Dispense from the valve	Mix	529–637
D5	Direct Dispense from the valve	Mix	10–672
D6	Direct Dispense from the valve	Mix	580–672

Grab samples (>50 ml) were collected at the manhole using a stainless-steel telescopic rod pole swivel dipper water swing sampler. Alternatively, samples were obtained from the valve by submerging a sterile Nalgene bottle into the flowing sewage. Sampling commenced at 8:00 am, and all collected samples were promptly transported to the BSL-2 laboratory in a cooler with ice. The transit time was kept to less than 3 h to ensure immediate processing.

Upon reaching the laboratory, sewage samples underwent pasteurization for 2 h at 60°C. Following pasteurization, centrifugation at 5,000 × *g* for 10 min occurred, and subsequent filtration was carried out through sequentially sized 0.45 and 0.22 μm nitrocellulose filters. These filters were individually placed in DNA LoBind tubes and stored at −80°C until DNA extraction. Concentration was achieved using an Amicon Ultra-15 filtration device, with centrifugation at either 4,000 × *g* for 30 min (Swing-arm rotor) or 5,000 x g for 20 min (Fixed-angle rotor) at room temperature. The resulting concentrated solution, approximately 250 μl, was carefully transferred to 2 ml DNA LoBind tubes.

RNA extraction was performed using the Qiagen viral RNA Mini Kit, following the instructions of the manufacturer, yielding 60 μl of extracted RNA, with a negative control using DNase/RNase-free water. Subsequently, the RNA samples were stored at −80°C and subjected to RT-qPCR analysis within 24 h following extraction (Ash et al., 2023; Li et al., 2023).

### RT-qPCR

To quantify the concentrations of SARS-CoV-2 and PMMoV RNA in each sample, we employed RT-qPCR. Specifically, we measured SARS-CoV-2 N1 using the TaqPath 1-Step RT-qPCR Master Mix, CG (Thermo Fisher Scientific) on an Applied Biosystems QuantStudios 7 Pro Real-Time PCR System instrument. Each 20 μl reaction mixture comprised 5 μl of 4X Master Mix (Thermo Fisher Scientific), 0.25 μl of a 10 μmol/L probe, 1 μl each of 10 μmol/L forward and reverse primers, 7.75 μl of nuclease-free water, and 5 μl of nucleic acid extract. After accurate pipetting of reagents into 96-well plates, a 10-s vortex mixing step followed. The RT-qPCR cycling conditions included an initial uracil-DNA glycosylase incubation for 2 min at 25°C, reverse transcription for 15 min at 50°C, activation of the Taq enzyme for 2 min at 95°C, and a two-step cycling process involving 3 s at 95°C and 30 s at 55°C, repeated for a total of 45 cycles. A positive test result was determined by the presence of an exponential fluorescent curve intersecting the threshold within 40 cycles (cycle threshold [Ct] < 40).

The quantification of PMMoV was executed using the TaqPath 1-Step RT-qPCR Master Mix, CG (Thermo Fisher Scientific) on a QuantStudios 7 Pro instrument. Each reaction was composed of 20 μl, including 5 μl of 4X Master Mix from Thermo Fisher Scientific, 0.5 μl of 10 μmol/L probe, 1.8 μl each of 10 μmol/L forward and reverse primers, 8.9 μl of nuclease-free water, and 2 μl of nucleic acid extract. The reagents were meticulously transferred into 96-well plates using pipettes and subsequently mixed by vortexing for 10 s. The thermocycling conditions utilized in this study were as follows: incubation of uracil-DNA glycosylase for 2 min at 25°C, reverse transcription carried out for 15 min at 50°C, activation of the Taq enzyme for 10 min at 95°C, and a two-step cycling process consisting of 30 s at 95°C followed by 1 min at 60°C, repeated for a total of 40 cycles.

In each RT-qPCR run, one positive PMMoV control and negative controls, comprising Mastermix and DNase/RNase-free water, were incorporated. The RT-qPCR reactions were carried out in triplicate, and the criteria for classifying a sample as positive included the requirement that all replicates produced positive results, with each individual replicate falling within the linear range of the standard curve. The N1 standard curve demonstrated a high level of efficiency, with a value of 94.669% (R2 = 1). The quantification of SARS-CoV-2 RNA was determined by calculating the average of three replicates of viral copies. The outputs of RT-qPCR were converted into units of copies per liter. In this study, the detection limit for SARS-CoV-2 and PMMoV was established at 20 and 10 copies per liter, respectively.

### DNA isolation, 16S rRNA gene amplification, sequencing

Before inclusion in the kit, quarter-sections of 0.45 μm and 0.22 μm nitrocellulose filters were prepared by flame-sterilizing a blade and using ethanol for sterilization. Genomic DNA extraction was then performed using the FastDNA Spin Kit for Soil (BIO101, Vista, CA, USA), strictly following the guidelines of manufacturer. Subsequent DNA purification utilized the SELECT-A-SIZE DNA Clean & Concentrator Kits (Zymo Research, Irvine, CA). The quality of the extracted DNA was assessed by determining the 260/280 and 260/230 ratios on a NanoDrop spectrophotometer (Thermo Fisher Scientific, Waltham, MA).

After confirming successful DNA extraction, Polymerase Chain Reaction (PCR) was conducted on 10 μl of the extracted DNA. DNA libraries were prepared following the methodology outlined by Caporaso et al. (2012). PCR amplification of the V4 region employed Phusion DNA polymerase (Master Mix; Thermo Fisher Scientific, Waltham, MA) and universal primers 515f and barcoded 806r, designed to anneal to both bacterial and archaeal sequences. A 12-bp barcode index on the reverse primer facilitated multiplexing for sequencing analysis.

Subsequently, amplicon quality and size were assessed using an Agilent Bioanalyzer (Agilent Technologies Santa Clara, CA). Following the protocol of manufacturer, the DNA amplicons were pooled and quantified with a NEBNext Library Quant Kit for Illumina (New England Biolabs, Ipswich, MA). Sequencing was performed using a MiSeq V2 kit on an Illumina MiSeq platform (Illumina, San Diego, CA).

Digital sequence data from the MiSeq underwent processing through the QIIME2 (v1.9) pipeline on a Linux Server (Caporaso et al., 2010). DADA2 within QIIME2 was employed for denoising, and fast-join facilitated the joining of paired-end sequences. Subsequent demultiplexing excluded sequences with a Phred score below 20, and UCHIME identified and removed chimeric sequences. Genus-level identification of sequences utilized the Silva database, with operational taxonomic units (OTUs) determined and sample populations normalized by total sequence count to ascertain the relative abundance of each OTU.

### Data analysis

Statistical analyses were conducted using R version 4.2.3. Initially, samples were processed by rarefying OTU tables to the lowest library size across all samples in each student residence hall. Subsequently, we computed common α-diversity metrics (Observed, ACE, Shannon, Simpson, InvSimpson, Fisher, Coverage, and PD) and α-diversity metric (Bray-Curtis) using the R phyloseq package. To assess differences in α-diversity metrics between groups, linear regression was employed, with semesters included as covariates. For the evaluation of differences in α-diversity metrics between groups, nonmetric-multidimensional scaling (NMDS) was utilized, and *p* values for the comparison between groups were determined using permutational multivariate ANOVA models, which included semesters as covariates. A Pearson Correlation analysis was undertaken to explore the correlations between various parameters, including the relative abundance of enteric pathogen and potential pathogens, the relative abundance of enteric pathogen and potential pathogen in positive and negative SARS-CoV-2 samples, as well as the relative abundance of potential pathogen in positive and negative SARS-CoV-2 sewage samples.

## Data Availability

The original contributions presented in the study are included in the article/Supplementary material, further inquiries can be directed to the corresponding author. The raw sequence reads are available here: https://www.ncbi.nlm.nih.gov/bioproject/PRJNA1260005.

## Supplementary material

SUPPLEMENTARY FIGURE S1 Histograms of linear discriminant analysis (LDA) effect size (LEfSe) comparison between positive and negative SARS-CoV-2 samples microbiota at the genus level in D3, D4, and D5. Log-level changes in LDA score are displayed on the x axis.
