# Supplementary figures and images for: SARS-CoV-2 virus in raw wastewater from student residence halls with concomitant 16S rRNA bacterial community structure changes
Source: Front Microbiol. 2025 Jun 2;16:1589029. doi: 10.3389/fmicb.2025.1589029 (PMC12171376; doi:10.3389/fmicb.2025.1589029)

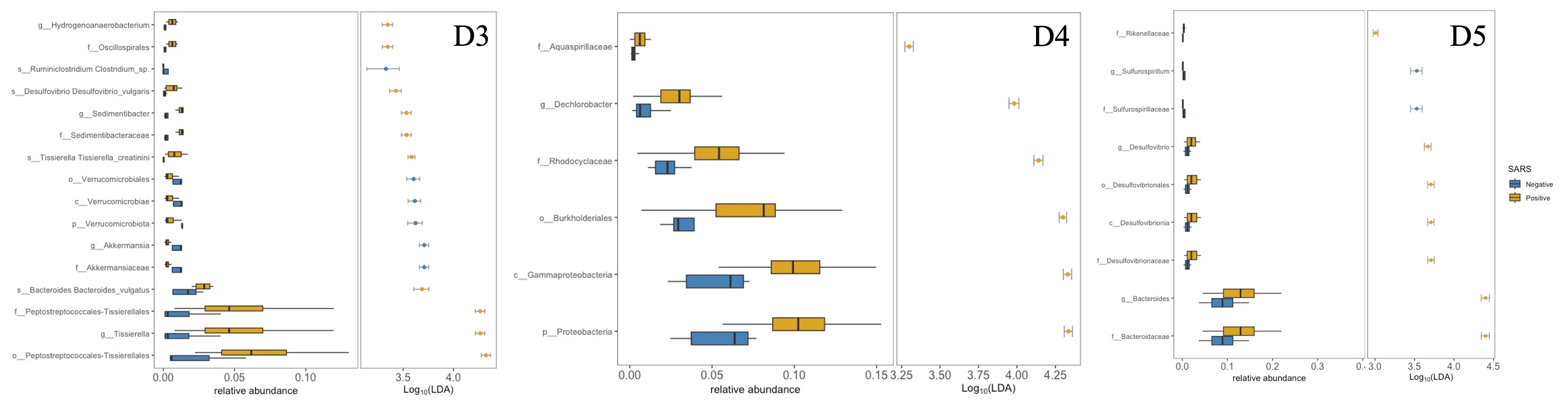

Supplement: SUPPLEMENTARY FIGURE S1 — Histograms of linear discriminant analysis (LDA) effect size (LEfSe) comparison between positive and negative SARS-CoV-2 samples microbiota at the genus level in D3, D4, and D5. Log-level changes in LDA score are displayed on the x axis. [file Supplementary_file_1.tif]
