# Supplementary material for: SARS-CoV-2 virus in raw wastewater from student residence halls with concomitant 16S rRNA bacterial community structure changes
Source: Front Microbiol. 2025 Jun 2;16:1589029. doi: 10.3389/fmicb.2025.1589029 (PMC12171376; doi:10.3389/fmicb.2025.1589029)
Supplement: Supplementary file 2 [file Supplementary_file_2.pdf]

https://www.ncbi.nlm.nih.gov/bioproject/PRJNA1260005

Accession	PRJNA1260005
Data Type	Raw sequence reads
Scope	Multispecies
Submission	Registration date: 7-May-2025<br>**University of Tennessee**
Relevance	Environmental
